# Validation of a Wireless Bluetooth Photoplethysmography Sensor Used on the Earlobe for Monitoring Heart Rate Variability Features during a Stress-Inducing Mental Task in Healthy Individuals

**DOI:** 10.3390/s20143905

**Published:** 2020-07-13

**Authors:** Bruno Correia, Nuno Dias, Patrício Costa, José Miguel Pêgo

**Affiliations:** 1 Life and Health Sciences Research Institute (ICVS), School of Medicine, University of Minho, 4710-057 Braga, Portugal; id7386@alunos.uminho.pt (B.C.); pcosta@med.uminho.pt (P.C.); 2 ICVS/3B’s - PT Government Associate Laboratory, 4710-057 Braga/Guimarães, Portugal; 3 iCognitus4ALL – IT Solutions, 4710-057 Braga, Portugal; 4 2Ai-Polytechnic Institute of Cávado and Ave, Campus do IPCA, 4750-810 Barcelos, Portugal; nuno.dias@mindproberlabs.com; 5 MindProber Labs, 4450-102 Porto, Portugal

**Keywords:** heart rate variability, pulse rate variability, photoplethysmography, heart rate signal, inter-beat intervals, James One, Polar H10, wearable devices

## Abstract

Heart rate variability (HRV), using electrocardiography (ECG), has gained popularity as a biomarker of the stress response. Alternatives to HRV monitoring, like photoplethysmography (PPG), are being explored as cheaper and unobtrusive non-invasive technologies. We report a new wireless PPG sensor that was tested in detecting changes in HRV, elicited by a mentally stressful task, and to determine if its signal can be used as a surrogate of ECG for HRV analysis. Data were collected simultaneously from volunteers using a PPG and ECG sensor, during a resting and a mentally stressful task. HRV metrics were extracted from these signals and compared to determine the agreement between them and to determine if any changes occurred in the metrics due to the stressful task. For both tasks, a moderate/good agreement was found in the mean interbeat intervals, SDNN, LF, and SD2, and a poor agreement for the pNN50, RMSSD|SD1, and HF metrics. The majority of the tested HRV metrics obtained from the PPG signal showed a significant decrease caused by the mental task. The disagreement found between specific HRV features imposes caution when comparing metrics from different technologies. Nevertheless, the tested sensor was successful at detecting changes in the HRV caused by a mental stressor.

## 1. Introduction

Heart rate variability (HRV) is the fluctuation over time of consecutive heartbeats and is accepted as a non-invasive biomarker of the activity of the autonomous nervous system [1,2,3]. The analysis of the HRV has been used as a diagnosis and a clinical research tool, since changes in HRV have been associated with several cardiovascular, metabolic, and mental disorders [2,3,4,5]. This marker has also shown potential for the monitoring of stress and pain responses and has been increasingly used in the sports field, as a tool to improve athletic performance [3,6,7,8,9,10].

The ECG signal is considered the gold standard from which the R-peaks from the QRS-complex can be identified using automatic computerized algorithms. The distance between these peaks is then used to create time-series of intervals between successive heartbeats (RR intervals) [4,11,12]. In clinical settings, these time records can go as far as 24 h, as they better reflect the natural fluctuations that occur on the body during the day. In the research field, a more straightforward approach is often taken, and most studies use data from five-minute (short-term) records. Time-domain, frequency-domain, and non-linear analysis are then used to extract different HRV metrics from these records [13].

PPG is a non-invasive optical method that allows the monitorization of the blood volume changes that occur on a micro vascularized tissue each time a heartbeat occurs [14,15]. Even though it was invented decades ago, this technique had a resurgence in recent years, as the need for smaller, simple, inexpensive, non-invasive methodologies for cardiovascular system assessment has increased, as well as major advances in a large array of technologies (e.g., optoelectrical components) and signal processing techniques [4,15]. This method has been used on various clinical applications like “cardiovascular system assessment, vital sign monitoring and blood oxygen detection.” [14].

The pulse wave signal generated from a PPG sensor and the pulse cycle intervals (PP intervals) extracted from it has been studied as a potential alternative to the ECG for HRV analysis, on what is frequently called pulse rate variability (PRV) analysis [4,12,14,16,17]. PPG sensors are generally cheaper, more readily available, and unobtrusive when compared to traditional or even portable ECG systems. These sensors can be easily placed on the wrist, finger, or ear, making them a hypothetically better alternative for the ambulatory monitorization of the HRV [4,12,14,16,17]. However, a consensus about the validity of the PRV as a surrogate of HRV has still not been reached. In their reviews, Schäfer and Vagedes [4] and Mejía et al. [16] explore this subject in detail, identifying studies were total, partial or no agreement was found between the compared metrics. These authors pointed out several technical and physiological factors that could impact the PRV analysis and in turn, explain the lack of agreement between PRV and HRV metrics. These factors include the sampling rate and the wavelength of the light used by the device, the fiducial point chosen to calculated the PP intervals, the processing technique used to obtain the frequency domain metrics, factors that have a negative impact in the signal quality (making harder the correct detection of the PP intervals) like motion artifacts, tone, and temperature of the skin, ambient light, and cardiovascular characteristics of the person. Another important factor is the time that it takes for the blood to travel from the heart to the place where the pulse wave signal is being recorded—the pulse transit time (PTT). The PTT can be influenced by several factors like the posture of the person when the signal is recorded, age, blood pressure, breathing rate, underlying heart or autonomic diseases, the execution of a physical or mental task, or even the sensor location (incongruences can be found in the PRV obtained from different body parts) [4,12,13,14,15,16,17,18,19,20,21].

The objective of this work is the validation of a recently released Bluetooth PPG sensor—James One (MindProber Labs, Porto, Portugal) for the ambulatory monitorization of a set of the most commonly used HRV metrics, that will be calculated from PP intervals provided by the sensor. As a reference device, we will use the Polar H10 (Polar Electro Oy, Kempele, Finland), a new iteration of the previously validated polar chest straps that makes use of ECG to provide RR intervals [1,3,6,11,22]. In this study, data will be acquired simultaneously using these two devices, and the agreement between the HRV features calculated from the RR and PP intervals will be determined. If an agreement is found, the James One PPG sensor can be used interchangeably with the Polar H10 for the ambulatory monitorization of the collection of selected HRV metrics. Furthermore, data will be collected during two different situations: at rest and through the execution of a mental stress-inducing task—the Stroop Color-Word Test (SCWT). The aim is to find if a mental stressor has an impact on the agreement between the data collected using these devices, as a reduced agreement has been reported during the execution of this kind of task [4]. The gathered data will also be used to determine if, regardless of the presence or absence of agreement among the HRV metrics obtained from two different technologies (ECG and PPG), the data obtained from the James One can still be used to detect changes that occur in HRV parameters instigated by the execution of a mentally stressful task.

## 2. Materials and Methods

### 2.1. Participants

Twenty-two volunteers were initially recruited. Exclusion criteria included the use of medication or any health condition that could induce abnormal changes in the heart rate signal. Data from four participants had to be excluded, as the collected signal had artifacts that would render the HRV analysis impossible, leaving our study with a total of 18 participants (age 31.1 ± 7.19 years old, 11 females and 7 males).

### 2.2. Software and Scripts Development

All the software and scripts developed for the data acquisition, visualization, processing, statistical analysis, and the computerized version of the SCWT were created using the Python programming language (Python Software Foundation, Version 3.7.4, available at https://www.python.org/) and the packages and modules that are part of the SciPy ecosystem [23,24,25,26,27].

### 2.3. Experimental Setup and Data Acquisition

**Figure 1:**
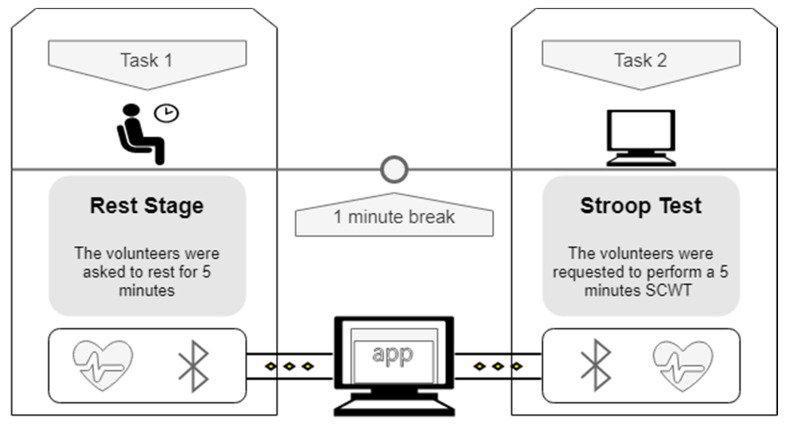
Representation of the experimental setup that was used for data acquisition. The experiment entailed two tasks with a duration of five minutes, separated by a one-minute break. Data was acquired and transmitted to a computer via Bluetooth low energy (BLE) using the Polar H10 and James One heart rate sensors, simultaneously.

The main steps of the experimental setup and data acquisition are represented in Figure 1. The experiment consisted of two five-minute tasks, separated by a one-minute break. Both tasks were performed with the participants in a sitting position. Participants could breathe freely. During the first task, the volunteers were asked to rest for five minutes. After a one-minute break, the participants were requested to perform a computerized version of the SCWT, developed specifically for the task. This test was used because it is easy to implement and has been shown to be an adequate mental stressor by previous studies, in which feelings of increased distress, heart rate, and the galvanic skin response have been reported [3,28,29,30].

**Figure 2:**
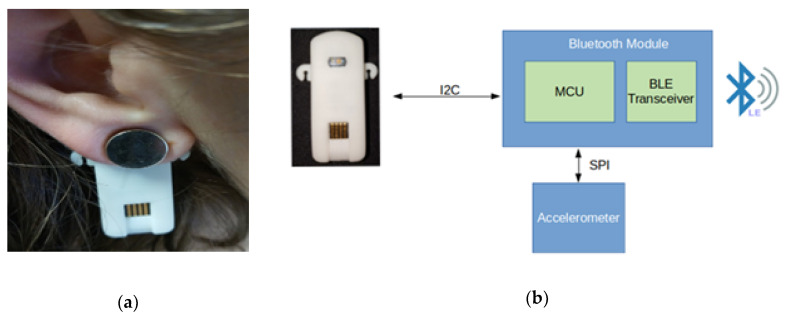
(**a**) James One held in place with a magnet on an earlobe. (**b**) Representation of the main components of the James One PPG sensor.

Data acquisition took place during both tasks and was performed simultaneously using a Polar H10 ECG sensor with the original Pro Strap and the James One PPG sensor (Figure 2). Both devices have a sampling rate of 1 kHz and proprietary algorithms embedded in their microcontrollers, that can detect when a heartbeat occurs, conveying directly via BLE the inter-beat intervals. To obtain the PP intervals, the James One sensor initially filters the PPG signal using a low-pass filter at 5 Hz (Butterworth, 2nd order) to avoid high-frequency noise. Its interval detection algorithm is applied to the first derivative of the filtered PPG signal to avoid baseline drifts. Then, the derivative signal is squared to increase the signal-to-noise ratio. The intervals are detected between derivative peaks, which are identified as the maximum values of the squared derivative signal above a dynamic threshold.

Following the guidelines provided in the Polar H10 instructions manual, the electrode area of the strap was moistened to improve signal acquisition and was adjusted under the chest. To minimize movement artifacts, the James One sensor was placed in the left earlobe, held in place with a magnet. The data from both devices was transmitted via BLE to a computer containing a software application specifically developed for the task. This application includes a graphical user interface and allows data to be recorded, timestamped, and visualized in real-time.

### 2.4. Data Processing

#### 2.4.1. Intervals Synchronization

Although the same application was used to receive and timestamp the PP and RR intervals, real-time synchronization of the data was not possible. One of the possible factors for this asynchronization is the different way these devices transmit data using the BLE protocol. Every second, the Polar H10 sends an array that contains the RR intervals that occurred in that time window. On the other hand, James One sends a PP interval each time a heartbeat occurs. This, allied with other uncontrollable factors, like potential delays in data transmission, the fact that BLE protocol does not provide timestamped intervals (data is timestamped when it is received on the acquisition software), and the delay originated from the different nature of the signals (PTT), makes it necessary to align the intervals before data analysis takes place. A script was created that performs this task automatically, by finding the position where the minimum variance and the maximal cross-correlation between the two time-series (RR and PP intervals) are present.

#### 2.4.2. Artifacts Correction

**Figure 3:**
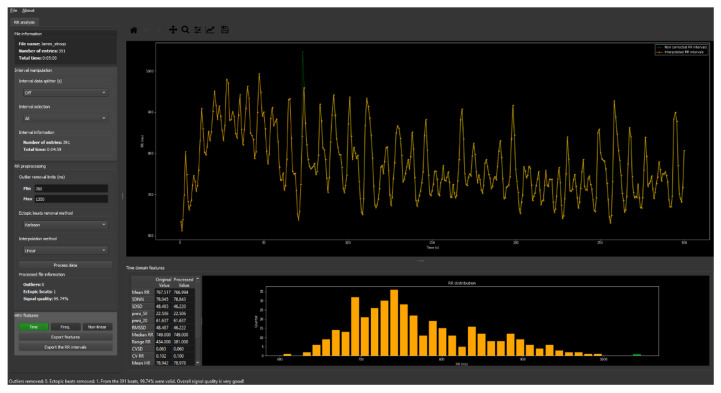
The interface of the software developed for the detection and correction of abnormal intervals.

Software was developed to detected and correct potential artifacts, either originated from technical problems (e.g., motion artifacts) or from a physiological origin (e.g., ectopic beats), in the obtained intervals to improve the overall quality of the HRV analysis. A PP or RR interval would be considered abnormal if its value was outside the 350–1350 milliseconds range or if it deviated 20% from the mean of the preceding and the subsequent interval [31,32]. Following other studies’ recommendations, abnormal intervals were replaced with values interpolated from adjacent intervals using linear interpolation (Figure 3) [2,32,33].

#### 2.4.3. HRV Features Calculations

Time and frequency domain and non-linear HRV measurements were calculated from the PP and RR intervals of each participant. The following time-domain features were used to quantify the amount of HRV in the five-minute time windows (short-term measurement): the mean of the intervals, the standard deviation of the intervals (SDNN), the percentage of adjacent intervals that differ more than 50 milliseconds (pNN50), and the root mean square of successive differences between heartbeats (RMSSD) “obtained by first calculating each successive time difference between heartbeats in ms. Then, each of the values is squared and the result is averaged before the square root of the total is obtained.” [13]. All the chosen time-domain features are expressed in milliseconds, except for the pNN50, which is represented in percentage [1,2,3,13,34]. To obtain the frequency–domain features, power spectral density was estimated using the Lomb–Scargle periodogram method. Only two of the three distinguishable core spectral components were used as features: the low frequency (LF; 0.04–0.15 Hz) and the high frequency (HF; 0.15–0.4 Hz) bands. The very-low-frequency band (0.003–0.04 Hz) was not included since short-term records were used. All the frequency domain measures are expressed in milliseconds squared (ms^2^) [1,2,3,13,35]. For the non-linear HRV analysis, where the unpredictability of the five-minute records is evaluated, scatter plots were created by plotting the current interval against the next interval—Poincaré plot. An ellipse centered on the average of the intervals was plotted. From this ellipse, the following HRV parameters were extracted: standard deviation 1 (SD1) that represents the ellipse width and standard deviation 2 (SD2) that represents its length. All the non-linear metrics are expressed in milliseconds [13,36]. Some studies use RMSSD and SD1 as independent metrics, apparently unaware that one can be obtained from the other using a constant multiplication. Even though these metrics produce similar results, we decided to include both, but we will refer to these two metrics as (one) RMSSD|SD1 in the discussion [37].

### 2.5. Data and Statistical Analysis

#### 2.5.1. Interbeat Intervals Description

To get a better understanding of the obtained intervals using the James One and the Polar H10 during the execution of the required tasks, all the participants’ intervals were pooled together and spread into four groups, called James One—Rest, Polar H10—Rest, James One—SCWT, and Polar H10—SCWT. For each of these groups, a histogram with the intervals was plotted, and descriptive statistics were performed.

#### 2.5.2. Determination of the Agreement between HRV Features

For the analysis of the agreement between the HRV features extracted from the intervals we followed a mixed approach and combined a set of different methods to determine the agreement.

First, we compared the relative variability between the HRV metrics extracted from the intervals using the James One and the Polar H10. This comparison was performed using the differences between the coefficients of variation (CV) calculated from the means and standard deviations (SD) of each pair of HRV features (e.g., SDNN of James One at rest is paired with SDNN of Polar H10 at rest for comparisons).

As a measure of effect size, we included Cohen’s d. This value was calculated using the differences between the means for each pair of HRV metrics divided from their pooled SDs. Since we had a small sample size, we had to apply a correction factor, in what is normally called the Hedges’s g (H_g_). Using Cohen’s guidelines (that depend on the situation), an H_g_ value equal to 0.2 represents a small effect, 0.5 a medium effect, and 0.8 a large effect [38,39].

Lin’s Concordance Correlation Coefficient (LCCC) was used to measure the agreement between the HRV metrics when normal distribution was present (Shapiro–Wilk test, *p* > 0.05) [40,41]. The following criteria for the interpretation of the LCCC was proposed by McBride [42]: almost perfect agreement if LCCC > 0.99, substantial agreement if 0.99 > LCCC > 0.95, moderate agreement if 0.95 > LCCC > 0.90, and poor agreement if LCCC < 0.9 [41,42]. However, and since these are merely a suggestion, we applied a more conservative approach, and considered that there was a poor agreement if LCCC < 0.95.

Another recommended procedure to assess the agreement between two methods is the Bland–Altman analysis [4,43,44,45]. This method does not require that the measurements obtained from the methods follow a normal distribution, but it assumes that the differences between the measures do [44,45]. Not all the differences between the HRV metrics obtained followed a normal distribution (Shapiro–Wilk test, *p* < 0.05). Even though a non-parametric approach is described by Bland et al. and used by the British Hypertension Society, its implementation was not possible as we could not find reference values for the maximum acceptable values to build the limits for each of HRV metric [40,45,46]. Nevertheless, Bland et al. suggest that “a non-normal distribution of differences may not be as serious here as in other statistical contexts”, so we decided to include the parametric approach of the Bland–Altman analysis [45]. In this approach, the average of the HRV metrics from both devices was plotted against the difference between methods. The mean of the differences between measurements, also known as bias or systematic error, was calculated and plotted, as well as the associated upper and lower limits of agreement (LoA, bias ± (1.96 × SD of the bias)) and the associated confidence intervals (CI, 95%) [43,44,45]. The Bland–Altman ratio (BA ratio) was calculated dividing half the range of the LoA by the mean of the pair of means of each HRV metric. Some authors suggest that a ratio lower than 0.1 indicates a good agreement, values higher than 0.1 and lower than 0.2 a moderate agreement, and higher than 0.2 a poor agreement [13,34,47].

An additional employed strategy was to plot all the pairs of HRV metrics against a 45° line (that would represent a perfect agreement) to visually evaluate how they deviate from it.

#### 2.5.3. Comparison of HRV Metrics during Rest and SCWT

To determine if James One could be used to detect changes in the HRV metrics caused by a mental stressor, we compared the data obtained from the resting task with the one from the SCWT. This comparison was also performed with the data obtained from the Polar H10, making a total of two comparison groups: (1) James One—Rest vs SCWT; and (2) Polar H10—Rest vs SCWT.

All the time and frequency domain features described in Section 2.4.3 were used. The use of absolute values for HF and LF alone can lead to an incorrect interpretation of results, so we also included the LF/HF ratio [30,48]. Only the nonlinear feature SD2 was included as using both RMSSD and SD1 is redundant [37].

Box plots were used to display the calculated HRV features (Rest vs SCTW). Each box plot is divided by a bar that represents the median (50th percentile). The spaces between the middle bar and the top and the bottom of the box indicate the 75th and 25th percentiles, respectively. The interquartile range (IQR) is the distance between the 75th and 25th percentiles. The whiskers that extend from the boxes represent the maximum (75th + 1.5 × IQR) and the minimum (25th − 1.5 × IQR) limits, in which a value is not regarded as an outlier. Values plotted outside these whiskers are considered outliers and are shown as ♦. Wilcoxon signed rank was used to compare the HRV metrics, as not all of them had a normal distribution. A *p* < 0.05 was considered significant. As a measure of effect size, we included the common-language effect size (CLES), that will tell us “the probability that a score sampled at random from one distribution will be greater than a score sampled from some other distribution.” [49].

## 3. Results

All the participants completed the required tasks. After the data was processed, four of the 22 participants had to be excluded due to anomalies (signal artifacts and abnormal PP/RR intervals) in the collected data that would render the HRV analysis and comparison impossible. Two of the participants had one gap at the end of their records using the James One, which could not be fixed with interpolation, so these gaps and the corresponding RR intervals obtained from the Polar H10 were removed so both data records could have the same length. For one of the participants, this gap was located at the end of task 1 (rest) and accounted for 20 s. The other’s participants’ gap was present at the end of task 2 (SCWT) and accounted for 30 s. From the 18 participants, a total of 14,296 intervals were obtained using the devices during the required tasks, 6976 at rest, and 7320 during the SCWT.

As shown in Table 1, the developed software detected 12 (0.17%) and 7 (0.1%) abnormal intervals in the James One and Polar H10 recordings during the task 1 (rest), respectively. During task 2 (SCWT), 9 (0.12%) errors were found in the James One intervals and 2 (0.03%) in the Polar H10.

**Figure 4:**
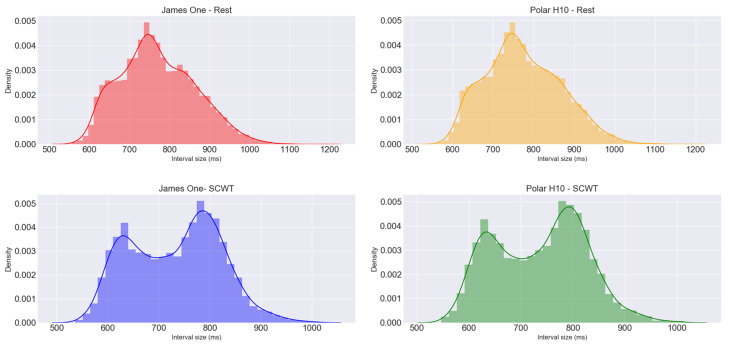
Histograms with a fitted kernel density estimation of the intervals obtained from the devices at rest and during the SCWT.

The minimum, maximum, mean, SD, skewness, kurtosis, and the CVs of the obtained PP and RR intervals are reported in Table 2. Both devices displayed similar CVs during the proposed tasks. The histograms in Figure 4 show the density of the distribution of the intervals from both devices during the required tasks. The intervals acquired at rest appear to have a unimodal distribution while during the SCTW the plot gives the idea of a bimodal distribution.

### 3.1. Agreement of the HRV Metrics at rest

**Figure 5:**
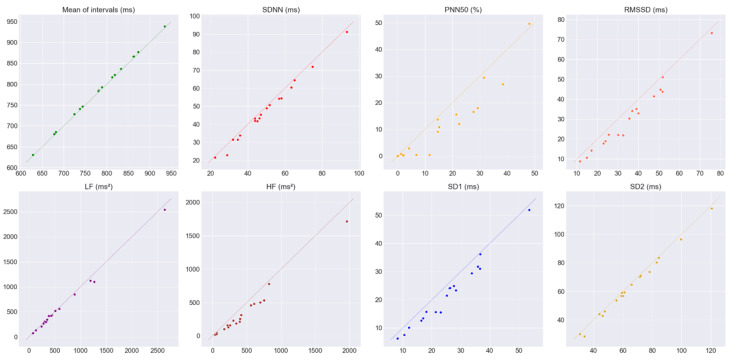
Pairs of HRV metrics obtained at rest plotted against a 45° line (that would represent a perfect agreement). The HRV metrics obtained from the James One are represented on the *x*-axis and the ones from the Polar H10 on the *y*-axis.

**Figure A1:**
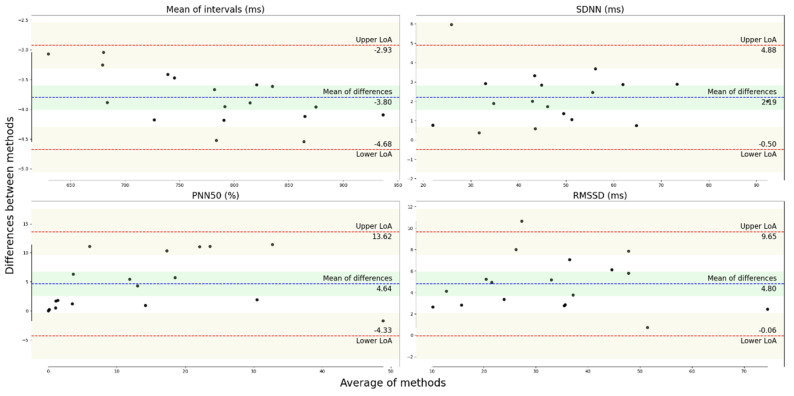
Bland–Altman plots for the pairs of time-domain metrics obtained at rest. The blue line and the green area represent the mean of the differences between measurements and the associated confidence interval. The red lines and the associated area represent the upper and lower LoAs and the associated confidence intervals.

**Figure A2:**
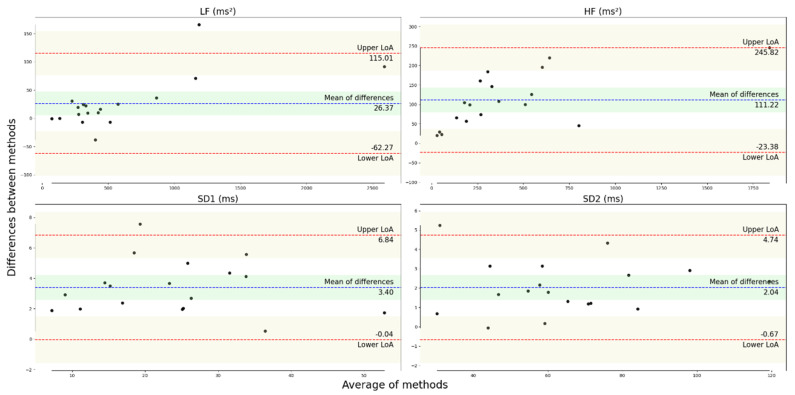
Bland–Altman plots for the pairs of frequency-domain and non-linear metrics obtained at rest. The blue line and the green area represent the mean of the differences between measurements and the associated confidence interval. The red lines and the associated area represent the upper and lower LoAs and the associated confidence intervals.

Table 3 shows the mean and the associated SD, differences between the CVs of the James One and the Polar H10, H_g_ value, LCCC, the mean of the differences between measurements (bias) and the associated SD, LoA from the Bland–Altman analysis, and the BA ratio for all the pairs of HRV metrics obtained at rest. The criteria used to interpret the data are also shown. Figure 5 shows the pair of HRV metrics at rest plotted against a 45° line (that would represent a perfect agreement). The Bland–Altman plots are shown in Figure A1 and Figure A2 (Appendix A).

### 3.2. Agreement of the HRV Metrics during the SCWT

**Figure 6:**
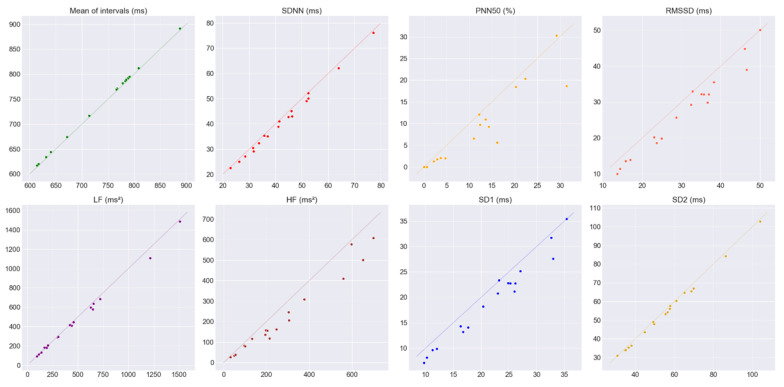
Pairs of HRV metrics obtained during the SCWT plotted against a 45º line (that would represent a perfect agreement). The HRV metrics obtained from the James One are represented on the *x*-axis and the ones from the Polar H10 on the *y*-axis.

**Figure A3:**
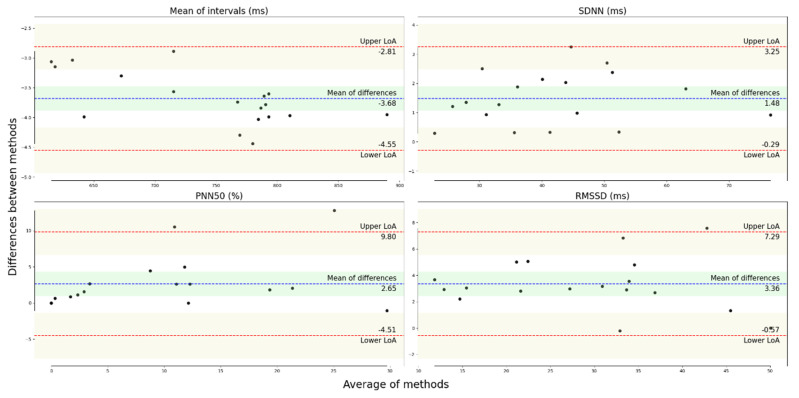
Bland–Altman plots for the pairs of time-domain metrics obtained during the SCWT. The blue line and area represent the mean of the differences between measurements and the associated confidence interval. The red lines and the associated area represent the upper and lower LoA and the associated confidence intervals.

**Figure A4:**
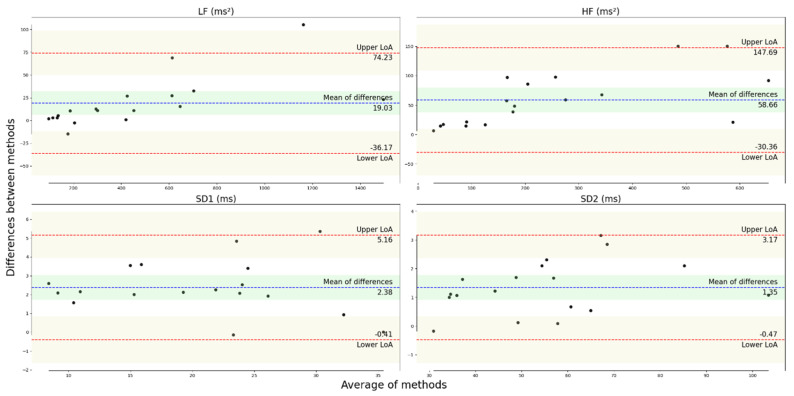
Bland–Altman plots for the pairs of frequency-domain and non-linear metrics obtained during the SCWT. The blue line and area represent the mean of the differences between measurements and the associated confidence interval. The red lines and the associated area represent the upper and lower LoA and the associated confidence intervals.

Table 4 shows the mean and the associated SD, differences between the CVs of the James One and the Polar H10, H_g_ value, LCCC, the mean of the differences between measurements (bias) and the associated SD, LoA from the Bland–Altman analysis, and the BA ratio for all the pairs of HRV metrics obtained during the SCWT. The criteria used to interpret the data are also shown. Figure 6 shows the pair of HRV metrics during the SCWT plotted against a 45° line (that would represent a perfect agreement). The Bland–Altman plots are shown in Figure A3 and Figure A4 (Appendix A).

### 3.3. Comparison between HRV Metrics at Rest and SCWT

**Figure 7:**
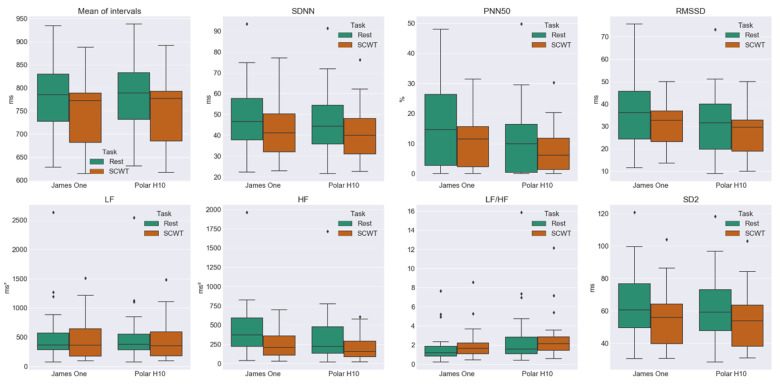
Box plots of the comparison between the HRV metrics obtained during the rest task and the SCWT. Each boxplot is divided by a bar that represents the median (50th percentile). The spaces between the middle bar and the top and the bottom of the box indicate the 75th and 25th percentiles, respectively. The IQR is the distance between the 75th and 25th percentiles. The whiskers that extend from the boxes represent the maximum (75th + 1.5 × IQR) and the minimum (25th − 1.5 × IQR) limits, in which a value is not regarded as an outlier. Values plotted outside these whiskers are considered outliers and are shown as ♦.

Figure 7 and Table 5 show the results of the comparison between the HRV metrics obtained during the rest task and the SCWT. Overall, there was a decrease in all of the metrics for both devices when comparing the values obtained from the resting task with the values from the SCWT, except for the LF/HF ratio, which increased in James One. For James One, this decrease was significant for the mean intervals, SDNN, pNN50, RMSSD, HF, and SD2. As for the Polar H10, the decreases were significant for the mean intervals, SDNN, HF, and SD2.

## 4. Discussion

The need for unobtrusive, simple, inexpensive methodologies for cardiovascular system monitoring, as well as the recent boom in the wearables market, has made the PPG methodology resurface in the last years [3,4,10,15,50]. Even though several commercially available wearable devices can monitor the heart rate signal using the PPG technology, they present one or several of the following drawbacks: low quality of the acquired data, lack of access to the data or no information of the used algorithms to process the data, privacy concerns (e.g., data sold to third party companies), high cost, proprietary software required to obtain the data, and technical problems [3,50,51,52]. The James Ones, however, has a low price and it allows direct access to its data, which can be obtained using standard BLE protocols giving the developers the freedom to create their algorithms for data processing and analysis.

The pulse wave signal generated by the PPG technology has been studied as a potential surrogate of the ECG signal for HRV analysis, but a consensus about its validity has an alternative has still not been reached. At resting conditions and using young and healthy participants, the available studies propose that this technology is a viable alternative, while others suggest that this technology as a tendency to overestimate some short-term variability metrics (pNN50, RMSSD, and HF) [4]. Several technical and physiological factors, on which the execution of mentally stressful tasks is included, have been suggested as potential sources of disagreement between the PRV and HRV metrics [4,16].

In our work, we aimed to validate for the first time the HRV measurements calculated from the PP intervals obtained from the heart rate sensor, James One, under two different conditions, at rest and during a mental stress-inducing task. As a reference, we used the RR intervals provided by a Polar H10 chest strap, an improved version of previously validated heart rate chest straps [1,3,6,11,22]. Regardless of the presence or absence of agreement between the HRV metrics obtained from these two devices, we tried to determine if the data extracted from the James One could be used to detect changes caused by the execution of a mentally stressful task.

Overall, the signal obtained from the James One and the Polar H10 appears to be good for both tasks, as a low number of abnormal intervals were detected. This was expected for the data collected from the Polar chest straps, where high signal quality is reported, even on physically demanding activities [1,11,22]. The motion generated artifacts are one of the most common problems in the pulse wave signal, but other factors like skin tone or temperature can hurt the signal quality [4,15,19,20]. Due to the nature of our experiment (lack of physical exertion), alike experimental conditions (e.g., similar skins tones and ambient temperatures), and the location of the sensor (left earlobe as opposed to the wrist or finger), this problem seems to have been mitigated.

Person’s Correlation Coefficients or mean comparisons (e.g., paired t-tests) are commonly used in the literature for the assessment of the agreement between the intervals and the HRV metrics obtained from two different devices. It is important to note however that the results obtained from these approaches can be highly misleading and their use to evaluate the agreement between methods has been deflated in previous works, where better alternatives are suggested (Bland–Altman analysis and Lin’s Concordance Correlation Coefficient) [4,41,43,44,45]. The use of these (occasionally wrong) methodologies makes the comparison of the results between articles hard, as sometimes the authors suggest that there is a good agreement between methods when in fact, there is only a good correlation. As we mentioned in our methodology, we decided to combine different methodologies to study the agreement between the HRV features extracted from the PP and RR intervals as we felt that a single simple approach could give misleading results.

At rest task and looking at the plots of the pairs of HRV metrics in Figure 5, it is possible to see that some features like the pNN50, RMSSD|SD1, and HF are overestimated by the James One, in comparison to the correspondent features calculated from Polar H10. The SDNN and SD2 seem to be slightly overestimated, the mean intervals somewhat underestimated, and the LF (although higher values look slightly overestimated) appears to fit the 45°. These observations are corroborated by the values of the bias, reported in Table 3. The mean intervals, LF and SD2 display small differences in their CVs, low H_g_ values, and, when possible to calculate, LCCC values that represent almost perfect agreement, and BA ratios that report a good agreement (except for the LF feature that indicates a moderate agreement). The pNN50 and HF metrics appear to have considerable differences in the CVs and H_g_ values. Their BA ratios seem to indicate a poor agreement. Although not high as the previous metrics, the differences in the CVs of the RMSSD|SD1 are still relevant when compared to the values from the mean intervals, LF, and SD2 metrics. This metric also shows an H_g_ value higher than 0.2. Its LCCC value suggests a poor agreement while its BA ratio a moderate agreement. The SDNN metric shows relatively low differences in the CVs, a small but still considerable H_g_ value, substantial to a moderate agreement in its LCCC, and a good agreement in its BA ratio. Taking all of this into account we considered that the mean intervals, LF, and SD2 metrics show good agreement between the James One and Polar H10. The SDNN appears to have a moderate agreement and the pNN50, RMSSD|SD1, and HF a poor agreement.

Regarding the HRV metrics calculated from the PP and RR intervals obtained during the SCWT, is once again possible to see (using the plots in Figure 6) that the James One tends to overestimate the pNN50, RMSSD|SD1 and HF, slightly overestimate the SDNN and SD2, and marginally underestimate the mean intervals. Again, the LF band appears to fit the 45° (even though higher values look slightly overestimated). The values of the bias from Table 4 seem to support these observations. The mean intervals, SDNN, LF, and SD2 appear to have similarly low differences in their CVs, H_g_ values lower than 0.2, LCCC values that indicate a perfect agreement (when available), and BA ratios that suggest good agreement (except for the LF that is branded as a moderate agreement). Although not as noticeable as in the rest task, the pNN50, RMSSD|SD1, and HF show noticeable differences in their CVs (in comparison to the mean intervals, SDNN, LF, and SD2 metrics) and H_g_ values. The RMSSD|SD1 LCCC value suggests a poor agreement. The BA ratios show a poor agreement for the pNN50 and the HF, and a moderate agreement for the RMSSD|SD1. Taking all of this into account we considered that the mean intervals, SDNN, LF, and SD2 metrics show a good agreement between the James One and Polar H10 and a poor agreement for the pNN50, RMSSD|SD1, and HF metrics.

Our findings are in line with other studies that suggest a tendency for the overestimation of certain HRV metrics when using a PPG sensor, a disagreement between short-term variability parameters such as the pNN50, RMSSD|SD1, and HF, and an agreement in metrics like the mean intervals, SDNN, LF, and SD2 [4,16,53]. It is unlikely that the disagreement found between the metrics is due to technical factors, as the overall quality of the signal obtained from the James One appears to be good (as we have previously reported) and the sensor has a high sampling rate (1 kHz). The lack of agreement in the short-term variability metrics could be explained by the variations in the PTT caused by the unconstrained breathing rates of our participants, as suggested by Schäfer and Vagedes [4]. This idea is supported by other studies, like the one conducted by Chen et al. [53], where a comparison of the impact of different respiration modes on the agreement between PRV and HRV metrics was performed. They found that during paced breathing, almost all the tested metrics (mean intervals, SDNN, RMSSD|SD1, LF, HF, and SD2) had a moderate/good agreement. However, with intermittent breath holding, short-term variability metrics like the RMSSD|SD1 and HF had an insufficient agreement. They suggested that the maintenance of a steady breathing pace was translated into a reduction in the variation of the PTT, potentially decreasing the differences between the PRV and HRV parameters. They concluded that, regardless of the tested respiratory mode, parameters like means intervals, SDNN, LF, and SD2 showed a satisfactory agreement, as opposed to short-term metrics [53]. Weinschenk et al. [34] also reported stronger agreements between PRV and HRV metrics when the breathing conditions were controlled in comparison to spontaneous breathing rates. Although the execution of a mentally stressful task had an impact on the values of some of the tested HRV features (Table 5), the agreement between the metrics did not appear to suffer any alteration apart from the SDNN metric, which improved from a moderate to a good agreement. As previously shown in another study and has seen in our results, reporting RMSSD and SD1 is redundant, as both metrics show similar results [37].

As shown in Table 5 and Figure 7, the HRV metrics extracted from the PP intervals obtained from the James One seem to present adequate sensitivity to detect changes in the HRV caused by a mental stressor. Almost all the tested metrics had a decrease that was deemed significant (mean intervals, SDNN, pNN50, RMSSD, HF, and SD2). For these metrics, an average of CLES ≈ 0.63 indicates that there is a 63% chance that if a value is randomly taken from the rest task, it will be greater than a value randomly sampled from the SCTW task [49]. Although there was a decrease in the LF and an increase in the LF/HF ratio, these alterations were not significant. These results are in line with evidence from other studies that use mental stressors, where decreases in the heartbeat intervals, SDNN, pNN50, RMSSD, absolute values of HF and LF and increases of LF/HF ratios are reported [8,30,54,55]. Even though the data obtained from the Polar H10 indicates a decrease in all the tested metrics due to the execution of the SCWT, these changes were only significant for the mean intervals, SDNN, HF, and SD2 (CLES ≈ 0.62). Oddly enough, the LF/HF suffered a slight (non-significant) decrease. Interestingly, although all metrics with moderate to good device agreement present similar differences between tasks, the features pNN50 and RMSSD, poorly congruent among devices, seems to discriminate tasks clearly when calculated from James One.

It is worth noticing that a poor agreement among some of the tested metrics does not mean that the signal obtained from the James One sensor lacks quality or is devoid of use for the monitoring of changes in HRV metrics with poor concordance. This only tells us that these metrics cannot be used interchangeably with the ones obtained from the Polar H10. Furthermore, the poor agreement in some metrics seems to result from the nature of the signal itself, as other studies report similar results [4,16]. In fact, considering that anxiety and stress states have been described as having a decreasing effect on HRV, the metrics calculated from James One seem to be often more sensitive to the stress-induction of the SCWT task, as seen in the results of Table 5, were the James One was able to significantly detect changes in the pNN50 and RMSSD, whilst the Polar H10 was not [8]. Nevertheless, some features extracted from the intervals provided from the James One, like the mean intervals, LF, and SD2, and, to a certain degree, the SDNN, can be used interchangeably with the features obtained from the Polar H10, that uses ECG.

## 5. Conclusions

At rest and during the execution of the SCTW, there was good agreement between the mean intervals, LF, and SD2 metrics extracted from the PP intervals provided by the James One and the RR intervals obtained from the Polar H10. As for the SDNN metric, there was a good agreement during the SCTW and a moderate agreement at rest. Metrics that reflect short-term variability, like the pNN50, RMSSD|SD1, and HF appear to have a poor agreement between sensors and seem to be overestimated when using PP intervals, in comparison to the correspondent features calculated from RR intervals. The execution of a mental task did not appear to negatively affect the agreement.

As previously reported, some incongruence was observed between specific HRV metrics calculated from RR and PP intervals which imposes some caution when comparing HRV metrics calculated from different technologies. Nevertheless, the data extracted from James One could be successfully used to detect changes in the HRV caused by the execution of a mentally stressful task. The mental stressor caused a significant decrease in the mean intervals, SDNN, pNN50, RMSSD|SD1, HF, and SD2. Considering its low-cost and usage flexibility, the reported results suggest that James One may be a promising, yet robust, sensor for measuring stress induction through HRV metrics.

## Figures and Tables

**Table 1:** The number of intervals obtained using both devices during the required tasks and errors detected using the developed software.

	Number of Intervals	Errors in the Intervals (%)	
		James One	Polar H10
Rest	6976	12 (0.17)	7 (0.10)
SCWT	7320	9 (0.12)	2 (0.03)
Total	14,296	21 (0.15)	9 (0.06)

**Table 2:** Descriptive statistics of the intervals obtained from the James One and Polar H10 at rest and during the SCWT.

	Nº. of Intervals	Min. (ms)	Max. (ms)	Mean (ms)	SD (ms)	Skewness	Kurtosis	CV (%)
James One—Rest	6976	555	1180	770	94	0.34	−0.33	12.2
Polar H10—Rest	6976	563	1180	774	93	0.34	−0.31	12.0
James One—SCWT	7320	536	1012	732	88	0.042	−0.81	12.1
Polar H10—SCWT	7320	547	1013	736	88	0.039	−0.82	11.9

**Table 3:** Mean and the associated SD, differences between the CVs of the James One (CV_J_) and the Polar H10 (CV_P_), H_g_ value, mean differences (bias) and the associated SD, for all the pairs of HRV metrics obtained at rest. Using Cohen’s guidelines, an H_g_ value equal to 0.2 represents a small effect, 0.5 a medium effect, and 0.8 a large effect [38,39]. The agreement between the parameters is shown as LCCC. Values higher than 0.99 represent an almost perfect agreement, substantial and moderate agreement when lower than 0.99 and higher than 0.95, and a poor agreement if lower than 0.95 [41,42]. BA ratios lower than 0.1 represent a good agreement, higher than 0.1 and lower than 0.2 represent a moderate agreement, and higher than 0.2 a poor agreement [13,34,47].

	Mean ± SD James One	Mean ± SD Polar H10	CV_J_ – CV_P_ (%)	H_g_	LCCC	Bias ± SD	LoA Upper; Lower	BA Ratio
Mean int. (ms)	778 ± 80.8	782 ± 81.1	0.015	−0.046	0.998	−3.80 ± 0.45	−2.93; −4.68	0.005
SDNN (ms)	49.6 ± 17.3	47.4 ± 17.4	−1.70	0.123	0.989	2.19 ± 1.38	4.88; −0.50	0.055
pNN50 (%)	16.2 ± 14.3	11.5 ± 13.4	−27.0	0.327	-	4.64 ± 4.58	13.6; −4.33	0.648
RMSSD (ms)	35.8 ± 15.9	31.0 ± 16.2	−7.34	0.292	0.944	4.80 ± 2.48	9.65; −0.060	0.145
LF (ms^2^)	593 ± 606	566 ± 575	0.64	0.0436	-	26.4 ± 45.2	115; −62.3	0.153
HF (ms^2^)	462 ± 443	350 ± 398	−17.1	0.2581	-	111 ± 68.7	246; −23.4	0.331
SD1 (ms)	25.4 ± 11.3	21.9 ± 11.4	−7.34	0.292	0.944	3.40 ± 1.75	6.84; −0.04	0.145
SD2 (ms)	65.1 ± 22.7	63.1 ± 23	−1.00	0.088	0.994	2.04 ± 1.38	4.74; −0.67	0.042

**Table 4:** Mean and the associated SD, differences between the CVs of the James One and the Polar H10, H_g_ value, mean differences (bias) and the associated SD, for all the pairs of HRV metrics obtained during the SCWT. Using Cohen’s guidelines, an H_g_ value equal to 0.2 represents a small effect, 0.5 a medium effect, and 0.8 a large effect [38,39]. The agreement between the parameters is shown as LCCC. Values higher than 0.99 represent an almost perfect agreement, substantial and moderate agreement when lower than 0.99 and higher than 0.95, and a poor agreement if lower than 0.95 [41,42]. BA ratios lower than 0.1 represent a good agreement, higher than 0.1 and lower than 0.2 represent moderate agreement, and higher than 0.2 a poor agreement [13,34,47].

	Mean ± SD James One	Mean ± SD Polar H10	CV_J_ – CV_P_ (%)	H_g_	LCCC	Bias ± SD	LoA Upper; Lower	BA Ratio
Mean int. (ms)	741 ± 77.5	744 ± 77.8	0.013	−0.046	-	−3.68 ± 0.44	−2.81; −4.55	0.005
SDNN (ms)	42.5 ± 13.7	41.0 ± 13.6	−0.85	0.106	0.992	1.48 ± 0.90	3.25; −0.29	0.042
pNN50 (%)	10.9 ± 10.1	8.3 ± 8.8	−13.8	0.274	-	2.65 ± 3.65	9.80; −4.51	0.744
RMSSD (ms)	30.7 ± 11.3	27.3 ± 11.5	−5.16	0.289	0.941	3.36 ± 2.01	7.29; −0.57	0.136
LF (ms^2^)	463 ± 389	444 ± 370	0.51	0.049	-	19.0 ± 28.2	74.2; −36.2	0.121
HF (ms^2^)	279 ± 214	220 ± 186	−7.14	0.286	-	58.7 ± 45.4	148; −30.4	0.357
SD1 (ms)	21.7 ± 7.97	19.3 ± 8.1	−5.16	0.289	0.941	2.38 ± 1.42	5.16; −0.405	0.136
SD2 (ms)	55.6 ± 18.9	54.3 ± 18.7	−0.31	0.070	0.996	1.35 ± 0.93	3.17; −0.47	0.033

**Table 5:** Comparison of the HRV metrics obtained during the resting stage and the SCWT. A ↓ indicates a decrease in the HRV metrics and ↑ an increase. A *p*-value lower than 0.05 indicates that the differences between the rest and the SCTW were significant. The effect size is reported as CLES.

	Mean int.	SDNN	pNN50	RMSSD	LF	HF	LF/HF	SD2
James One Rest → SCWT	↓; *p* = 0.002 CLES = 0.633	↓; *p* = 0.005 CLES = 0.623	↓; *p* = 0.008 CLES = 0.602	↓; *p* = 0.017 CLES = 0.611	↓; *p* = 0.082 CLES = 0.556	↓; *p* = 0.003 CLES = 0.651	↑; *p* = 0.408 CLES = 0.602	↓; *p* = 0.009 CLES = 0.633
Polar H10 Rest → SCWT	↓; *p* = 0.002 CLES = 0.636	↓; *p* = 0.015 CLES = 0.623	↓; *p* = 0.171 CLES = 0.528	↓; *p* = 0.055 CLES = 0.571	↓; *p* = 0.117 CLES = 0.571	↓; *p* = 0.008 CLES = 0.605	↓; *p* = 0.931 CLES = 0.556	↓; *p* = 0.017 CLES = 0.620

## References

[1] Caminal P., Sola F., Gomis P., Guasch E., Perera A., Soriano N., Mont L. (2018). Validity of the Polar V800 monitor for measuring heart rate variability in mountain running route conditions. Eur. J. Appl. Physiol..

[2] Malik M., Bigger J.T., Camm A.J., Kleiger R.E., Malliani A., Moss A.J., Schwartz P.J. (1996). Heart rate variability: Standards of measurement, physiological interpretation, and clinical use. Eur. Heart J..

[3] Hernando D., Roca S., Sancho J., Alesanco Á., Bailón R. (2018). Validation of the apple watch for heart rate variability measurements during relax and mental stress in healthy subjects. Sensors.

[4] Schäfer A., Vagedes J. (2013). How accurate is pulse rate variability as an estimate of heart rate variability? A review on studies comparing photoplethysmographic technology with an electrocardiogram. Int. J. Cardiol..

[5] Billman G.E., Huikuri H.V., Sacha J., Trimmel K. (2015). An introduction to heart rate variability: Methodological considerations and clinical applications. Front. Physiol..

[6] Plews D.J., Scott B., Altini M., Wood M., Kilding A.E., Laursen P.B. (2017). Comparison of heart-rate-variability recording with smartphone photoplethysmography, polar H7 chest strap, and electrocardiography. Int. J. Sports Physiol. Perform..

[7] Alberdi A., Aztiria A., Basarab A. (2016). Towards an automatic early stress recognition system for office environments based on multimodal measurements: A review. J. Biomed. Inform..

[8] Kim H.-G., Cheon E.-J., Bai D.-S., Lee Y.H., Koo B.-H. (2018). Stress and Heart Rate Variability: A Meta-Analysis and Review of the Literature. Psychiatry Investig..

[9] Ledowski T., Tiong W.S., Lee C., Wong B., Fiori T., Parker N. (2013). Analgesia nociception index: Evaluation as a new parameter for acute postoperative pain. Br. J. Anaesth..

[10] Dobbs W.C., Fedewa M.V., MacDonald H.V., Holmes C.J., Cicone Z.S., Plews D.J., Esco M.R. (2019). The Accuracy of Acquiring Heart Rate Variability from Portable Devices: A Systematic Review and Meta-Analysis. Sports Med..

[11] Gilgen-Ammann R., Schweizer T., Wyss T. (2019). RR interval signal quality of a heart rate monitor and an ECG Holter at rest and during exercise. Eur. J. Appl. Physiol..

[12] Pernice R., Javorka M., Krohova J., Czippelova B., Turianikova Z., Busacca A., Faes L. Reliability of Short-Term Heart Rate Variability Indexes Assessed through Photoplethysmography. Proceedings of the 2018 40th Annual International Conference of the IEEE Engineering in Medicine and Biology Society (EMBC).

[13] Shaffer F., Ginsberg J.P. (2017). An Overview of Heart Rate Variability Metrics and Norms. Front. Public Health.

[14] Sun Y., Thakor N. (2016). Photoplethysmography Revisited: From Contact to Noncontact, from Point to Imaging. IEEE Trans. Biomed. Eng..

[15] Allen J. (2007). Photoplethysmography and its application in clinical physiological measurement. Physiol. Meas..

[16] Mejía-Mejía E., May J.M., Torres R., Kyriacou P.A. (2020). Pulse rate variability in cardiovascular health: A review on its applications and relationship with heart rate variability. Physiol. Meas..

[17] Peralta E., Lazaro J., Bailon R., Marozas V., Gil E. (2019). Optimal fiducial points for pulse rate variability analysis from forehead and finger photoplethysmographic signals. Physiol. Meas..

[18] Yuda E., Yamamoto K., Yoshida Y., Hayano J. (2020). Differences in pulse rate variability with measurement site. J. Physiol. Anthropol..

[19] Castaneda D., Esparza A., Ghamari M., Soltanpur C., & Nazeran H. (2018). A review on wearable photoplethysmography sensors and their potential future applications in health care. Int. J. Biosens. Bioelectron..

[20] Khan M., Pretty C.G., Amies A.C., Elliott R., Shaw G.M., Chase J.G. (2015). Investigating the Effects of Temperature on Photoplethysmography. IFAC-PapersOnLine.

[21] Wong J.-S., Lu W.-A., Wu K.-T., Liu M., Chen G.-Y., Kuo C.-D. (2012). A comparative study of pulse rate variability and heart rate variability in healthy subjects. J. Clin. Monit. Comput..

[22] Giles D., Draper N., Neil W. (2016). Validity of the Polar V800 heart rate monitor to measure RR intervals at rest. Eur. J. Appl. Physiol..

[23] Van Rossum G., Drake F.L. (1995). Python Tutorial. Centrum voor Wiskunde en Informatica.

[24] Virtanen P., Gommers R., Oliphant T.E., Haberland M., Reddy T., Cournapeau D., Burovski E., Peterson P., Weckesser W., Bright J. (2020). SciPy 1.0: Fundamental Algorithms for Scientific Computing in Python. Nat. Methods.

[25] Hunter J.D. (2007). Matplotlib: A 2D Graphics Environment. Comput. Sci. Eng..

[26] van der Walt S., Colbert S.C., Varoquaux G. (2011). The NumPy Array: A Structure for Efficient Numerical Computation. Comput. Sci. Eng..

[27] (2020). Pandas-Dev/Pandas: Pandas 1.0.0. https://zenodo.org/record/3630805#.Xw0FmiMzaUk.

[28] Tulen J.H., Moleman P., van Steenis H.G., Boomsma F. (1989). Characterization of stress reactions to the Stroop Color Word Test. Pharmacol. Biochem. Behav..

[29] Karthikeyan P., Murugappan M., Yaacob S. (2014). Analysis of Stroop Color Word Test-Based Human Stress Detection using Electrocardiography and Heart Rate Variability Signals. Arab. J. Sci. Eng..

[30] Delaney J.P.A., Brodie D.A. (2000). Effects of short-term psychological stress on the time and frequency domains of heart-rate variability. Percept. Mot. Skills.

[31] Karlsson M., Hörnsten R., Rydberg A., Wiklund U. (2012). Automatic filtering of outliers in RR intervals before analysis of heart rate variability in Holter recordings: A comparison with carefully edited data. Biomed. Eng. Online.

[32] Peltola M.A. (2012). Role of editing of R–R intervals in the analysis of heart rate variability. Front. Physiol..

[33] Choi A., Shin H. (2018). Quantitative Analysis of the Effect of an Ectopic Beat on the Heart Rate Variability in the Resting Condition. Front. Physiol..

[34] Weinschenk S.W., Beise R.D., Lorenz J. (2016). Heart rate variability (HRV) in deep breathing tests and 5-min short-term recordings: Agreement of ear photoplethysmography with ECG measurements, in 343 subjects. Eur. J. Appl. Physiol..

[35] Ang S.-S., Wieczorkowski-Rettinger K., Hernandez-Silveira M., Carrara S., Iniewski K. (2015). Real-time activity energy expenditure estimation for embedded ambulatory systems using Sensium^TM^ technologies. Handbook of Bioelectronics.

[36] Stein P.K., Reddy A. (2005). Non-linear heart rate variability and risk stratification in cardiovascular disease. Indian Pacing Electrophysiol. J..

[37] Ciccone A.B., Siedlik J.A., Wecht J.M., Deckert J.A., Nguyen N.D., Weir J.P. (2017). Reminder: RMSSD and SD1 are identical heart rate variability metrics. Muscle Nerve.

[38] Turner I., Herbert M., Bernard R.M. (2006). Calculating and Synthesizing Effect Sizes. Contemp. Issues Commun. Sci. Disord..

[39] Cohen J. (1988). Statistical Power Analysis for the Behavioral Sciences.

[40] Shapiro S.S., Wilk M.B. (1965). An Analysis of Variance Test for Normality (Complete Samples). Biometrika.

[41] Lin L.I. (1989). A Concordance Correlation Coefficient to Evaluate Reproducibility. Biometrics.

[42] McBride G.B. (2005). A Proposal for Strength-of-Agreement Criteria for Lin’s Concordance Correlation Coefficient.

[43] Giavarina D. (2015). Understanding Bland Altman analysis. Biochem. Med..

[44] Bland J.M., Altman D. (1986). Statistical Methods for Assessing Agreement Between Two Methods of Clinical Measurement. Lancet.

[45] Bland J.M., Altman D.G. (1999). Measuring agreement in method comparison studies. Stat. Methods Med. Res..

[46] O’Brien E., Petrie J., Littler W., de Swiet M., Padfield P.L., Altman D., Bland M., Coats A., Atkins N. (1993). The British Hypertension Society protocol for the evaluation of blood pressure measuring devices. J. Hypertens..

[47] Radespiel-Tröger M., Rauh R., Mahlke C., Gottschalk T., Mück-Weymann M. (2003). Agreement of two different methods for measurement of heart rate variability. Clin. Auton. Res..

[48] Burr R.L. (2007). Interpretation of Normalized Spectral Heart Rate Variability Indices in Sleep Research: A Critical Review. Sleep.

[49] McGraw K.O., Wong S.P. (1992). A common language effect size statistic. Psychol. Bull..

[50] Nogueira P., Urbano J., Reis L.P., Cardoso H.L., Silva D.C., Rocha A.P., Gonçalves J., Faria B.M. (2018). A Review of Commercial and Medical-Grade Physiological Monitoring Devices for Biofeedback-Assisted Quality of Life Improvement Studies. J. Med. Syst..

[51] Yetisen A.K., Martinez-Hurtado J.L., Ünal B., Khademhosseini A., Butt H. (2018). Wearables in Medicine. Adv. Mater..

[52] Haghi M., Thurow K., Stoll R. (2017). Wearable Devices in Medical Internet of Things: Scientific Research and Commercially Available Devices. Healthc. Inform. Res..

[53] Chen X., Huang Y.-Y., Yun F., Chen T.-J., Li J. (2015). Effect of changes in sympathovagal balance on the accuracy of heart rate variability obtained from photoplethysmography. Exp. Ther. Med..

[54] Hjortskov N., Rissén D., Blangsted A.K., Fallentin N., Lundberg U., Søgaard K. (2004). The effect of mental stress on heart rate variability and blood pressure during computer work. Eur. J. Appl. Physiol..

[55] Melillo P., Formisano C., Bracale U., Pecchia L. Classification Tree for Real-Life Stress Detection Using Linear Heart Rate Variability Analysis. Case Study: Students under Stress Due to University Examination. Proceedings of the World Congress on Medical Physics and Biomedical Engineering.

